# Noncontinuous use of antidepressant in adults with major depressive disorders – a retrospective cohort study

**DOI:** 10.1002/brb3.224

**Published:** 2014-03-14

**Authors:** Wai-Yin Yau, Man-Chi Chan, Yun-Kwok Wing, Ho-Bun Lam, Wei Lin, Siu-Ping Lam, Chui-Ping Lee

**Affiliations:** 1 Department of Pharmacy, Prince of Wales HospitalShatin, Hong Kong; 2 Department of Psychiatry, Faculty of Medicine, The Chinese University of Hong KongShatin, Hong Kong; 3 Department of Psychiatry, Prince of Wales HospitalShatin, Hong Kong; 4 Faculty of Medicine, School of Pharmacy, The Chinese University of Hong KongShatin, Hong Kong

**Keywords:** Adherence, antidepressant, depression, pharmacotherapy, treatment

## Abstract

**Background:**

Noncontinuous antidepressant use is frequently observed in clinical practice despite the standard recommendation of at least 6–9 months of continuous treatment. The problem may be more serious in Chinese populations where stigmatization is common. This retrospective cohort study investigated the rate of noncontinuous antidepressant use and subsequent rate of relapse and recurrence in psychiatric Chinese outpatients by examining the prescription records, electronic medical records, and written medical records. Factors associated with noncontinuous antidepressant use were also identified.

**Methods:**

We reviewed the medical records of 189 patients newly dispensed with an antidepressant in the psychiatric outpatient clinic during year 2006 and 2007. Primary outcome was the rate of noncontinuous antidepressant use within 6 months of therapy. Secondary outcomes included the factors associated with noncontinuous antidepressant use and the rate of subsequent depression relapse and recurrence within 1 year of starting treatment.

**Results:**

Among the 189 subjects included in this study, 46% were noncontinuous users of the newly prescribed antidepressant therapy. The noncontinuous users were found to have an eightfold increase (OR: 8.42, 95% CI: 3.30–21.47) in the risks of relapse/recurrence depressive episodes within 1 year after treatment initiation. Younger age (*P* = 0.008), female, (*P* = 0.029), residency in public housing estate (*P* = 0.029), experiencing side effects (*P* = 0.024), infrequent follow-ups (*P* = 0.006), and earlier onset of diagnosis (*P* = 0.034) were factors significantly associated with noncontinuous antidepressant use.

**Conclusions:**

Noncontinuous antidepressant use is common in the local Chinese depressive patients and associated with a high rate of relapse and recurrence. Collaborative multidisciplinary approaches that target patient education and enhancement of follow-up adherence are needed.

## Introduction

Despite strong evidence available to support at least 6-month use of antidepressant to prevent relapse and recurrence, non-adherence or early discontinuation of antidepressants remained as a major treatment obstacle. A comprehensive review of papers published between 1975 and 2001 had previously reported that the early discontinuation rate was generally high, in which approximately 30% of patients stopped taking antidepressants within 1 month of commencing treatment and 45–60% stopped the prescribed treatment by 3 months (Lingam and Scott 2002). A more recent review of contemporary data published since 2001 found that the 6-month antidepressant non-adherence rate was 52% in the psychiatric population and 46.2% in the primary care population (Sansone and Sansone 2012). Indeed, the non-adherence rates found in the included studies ranged widely from 5.4 to 87.6% (Sansone and Sansone 2012). Apparently, the improved side-effect profiles of new medications did not resolve the issue of non-adherence.

The range of early non-adherence rate reported in these studies may be related to the inconsistent measures of non-adherence that were used, and the differences in culture and practices among different regions (Lingam and Scott 2002; Morgan et al. 2011; Wu et al. 2012). Of interest, the literature has suggested that depressive Chinese patients tend to deny depression, express symptoms somatically and emphasize self-management (Parker et al. 2001; Karasz 2005). In addition to underrecognition or misunderstanding of the presentation of depressive symptoms, Chinese patients have also been found reluctant to characterize their depressive symptoms as a psychiatric illness due to their culture's stigmatization of mental disorders (Yeung et al. 2004). In clinical practice, practitioners commonly encounter Chinese patients who believe that antidepressants only provide superficial and symptomatic relief with great liability of dependence. While negative attitudes toward depression and concerns over stigma have been reported to negatively affect medication adherence and help-seeking behaviors (Sirey et al. 2001a,b2001b), it remains unclear how the overall effect translates into actual non-adherence and adverse treatment outcomes in Chinese patients. Interestingly, a study conducted in Taiwan that examined the predictive values of self-stigma, insight, and perceived adverse effects of medication for remission of depressive symptoms found that degrees of self-stigma and insight did not necessarily predict the level of the medication adherence in their patient sample (Yen et al. 2009).

A number of studies have previously suggested that there may be increased risks of relapse or recurrence in patients who discontinued antidepressant prematurely (Claxton et al. 2000; Geddes et al. 2003; Kim et al. 2011; Lu and Roughhead 2012). A pooled analysis of four randomized, double-blinded, active comparator, 6-month trials in major depressive disorder also showed that there was a higher probability of achieving remission in the long term if 6-month treatment was completed (Wade et al. 2009). Most of these studies consisted of a mixture of psychiatrist-treated and non-psychiatrist-treated patients which may have also affected the treatment adherence rates (Akincigil et al. 2007; Lu and Roughhead 2012). However, the data were limited by utilization of only one source (e.g., claims data) to measure the adherence rate or medication possession rate.

### Aims of the study

Considering all of the above, we designed a retrospective cohort study to evaluate both the prescription record and also the electronic and written medical records of depressive patients to study the rate of noncontinuous antidepressant use in a psychiatric outpatient clinic setting in a Chinese population. We also investigated the association between noncontinuous use and relapse or recurrence within 1 year of starting treatment and the factors associated with noncontinuous antidepressant use.

## Material and Methods

### Study design, source of data, and patient population

This retrospective cohort study was conducted in the Prince of Wales Hospital (PWH) in Shatin, Hong Kong. The study was approved by the Joint Chinese University of Hong Kong and New Territories East Cluster (CUHK-NTEC) Clinical Research Ethics Committee (CREC) in July 2008. Eligible patients were identified through data retrieval using the Clinical Data Analysis and Reporting System (CDARS). This centralized-computerized database stores longitudinal clinical information including patient demographics, prescription, and dispensing records, and diagnosis patients received from all public hospitals in Hong Kong. Patients were included if (1) he or she was aged >18 years, (2) was attending Psychiatric Specialist Outpatient Clinic (SOPD) of PWH during the period between 1st January 2006 to 31st December 2007, (3) had an antidepressant dispensed during the study period, and (4) were diagnosed with major depressive disorders according to the International Classification of Disease-10 classification of mental and behavioral disorders. Subjects were excluded if they had (1) concurrent diagnosis of bipolar disorder, obsessive-compulsive disorder, psychosis, schizophrenia, substance abuse, post-traumatic stress disorder, acute stress disorder, and panic disorders, or (2) a diagnosis of dementia or mental retardation, or (3) a prescription of antidepressants 6 months prior to the study period, or (4) documented concurrent psychiatric-related follow-ups in other health care settings (where they may also had been prescribed antidepressants), or 5) history of drug overdose or suicide. If there were more than one period of antidepressant use during the study period, only the first episode was counted. Electronic patient records were then reviewed to confirm the eligibility. Further information regarding the antidepressant use and relapse and recurrence after treatment initiation was supplemented by reviewing the written medical records.

### Data collection

A standard form was designed for collecting patient-, illness- and treatment-related parameters. These included demographics such as age, gender, type of accommodation (as an indirect indictor for socioeconomic status) and marital status. Details of treatment regimen including drug name, dosage, frequency, duration, and prescription refill pattern were documented. Other treatment-related factors such as the frequency of follow-up were also recorded.

#### Definitions of continuous users, noncontinuous users, and relapse

In this study, continuous users were defined as patients who filled prescriptions for any antidepressants with no gaps of >15 days within 6 months after initiation of treatment. If there was a switch in antidepressants, there should also have been no extended breaks of >15 days in therapy (except when the switch involved monoamine oxidase inhibitors). Patients were considered noncontinuous users if (1) their prescriptions were filled with gaps of a total of >15 days within 6 months after treatment initiation among visits, or (2) or they had a history of documentation of noncontinuous use or non-adherence in medical records, and (3) did not return for follow-up, and did not have a documented change of care. The median time to medication noncontinuous use was calculated as the number of consecutive days from initiation of antidepressants to the start of the first medication gap. Reasons for the noncontinuous antidepressant use were documented.

All subjects were followed up for 1 year after treatment initiation to collect data on relapse and recurrence. Relapse was defined in this study as a psychiatrist-documented worsening of symptoms in a patient who has responded to treatment, while recurrence was defined as a return of symptoms in a patient who previously remitted (Frank et al. 1991).

### Outcome measurements and analysis

The primary outcome of the study was the percentage of subjects with noncontinuous antidepressant use. Secondary outcomes included the correlation between noncontinuous antidepressant use and relapse and recurrence of depressive episodes within 1 year after treatment, and correlations of various patient-related, treatment-related, and illness-related factors with the continuity of antidepressant treatment. The median time to noncontinuous use, mean dosage on discontinuation, and major reason for noncontinuous use were also assessed.

The Statistical Package for Social Science (SPSS v.13; SPSS Inc, Chicago, IL) was used for statistical analysis. The proportion of continuous and noncontinuous users was expressed in percentage and the median time to noncontinuous antidepressant use was expressed in number of days. Univariate analyses were performed using chi-square test to compare the categorical parameters between continuous and noncontinuous users. Two independent samples t-test were used for the comparison of age. Mann–Whitney Test was used to compare the continuous variables without a normal distribution (i.e., number of prescription and number of switch) between continuous and noncontinuous users. Variables with *P* < 0.2 in the univariate analyses were then analyzed by logistic regression. The effects on continuity of antidepressant treatment by different individual determinants were assessed by Odds Ratio (OR) and 95% Confidence intervals. The significance level was set at *P* < 0.05.

## Results

### Study population

A total of 355 patients newly prescribed with antidepressant during the study period were identified through data retrieval using CDARS. After reviewing the electronic patient records, 166 patients were excluded as they had other coexisting psychiatric conditions (*n* = 67, 40.4%), history of drug overdose or suicide attempts (*n* = 58, 34.9%), documented psychiatric follow-ups in other health care setting (*n* = 30, 18.1%), substance abuse (*n* = 9, 5.4%) or unavailability of written medical records (*n* = 2, 1.2%). Subsequently, 189 patients were included in the study for analysis. Table 1 showed the baseline demographic data of the subjects. The study sample was predominately female (71.4%). The mean age was 46.1 years (range: 20–88 years ). The majority (91.0%) of the patients had received primary education or above. The duration of their depressive illness ranged between 1 and 5 years (1.8 ± 0.7 years). Similar demographics of depressive patients have been reported in other studies conducted in Hong Kong (Lam et al. 2008; Li et al. 2012).

**Table 1:** Characteristics of 189 included study samples

Age (Mean years ± SD)	46.1 ± 14.8
Gender	
Female (%)	71.4
Male (%)	28.6
Living situation	
Living alone (%)	15.9
With others (%)	84.1
Type of accommodation	
Private (%)	46.7
Public (%)	53.3
Marital status	
Married (%)	50.3
Single (%)	19.0
Divorced/widow (%)	30.7
Smoking habit	
Ex-smoker (%)	9.1
Non-smoker (%)	60.8
Smoker (%)	15.3
Unknown (%)	14.8
Drinking habit	
Ex-drinker (%)	2.1
Nondrinker (%)	69.8
Drinker (%)	3.7
Social drinker (%)	11.6
Unknown (%)	12.8
Educational level	
Illiterate (%)	9.0
Primary (%)	25.0
Secondary (%)	48.4
Tertiary or above (%)	17.6
Duration of illness (mean years ± SD)	1.8 ± 0.69
Antidepressant started	
Mirtazapine (%)	4.2
SSRI (%)	71.4
TCA & its related AD (%)	12.1
SNRI (%)	8.5
Others (%)	3.8

### Continuity of treatment and association with relapses within 1 year of treatment

Out of 189 included subjects, 46.0% were noncontinuous users during the 6-month course of treatment (i.e., prescriptions were filled with gaps of a total of >15 days or had documentation of noncontinuous use). The rate of early noncontinuous antidepressant use within the first 30 days of treatment was 12.2% (*n* = 23). Noncontinuous users were significantly more prone to having a relapse or recurrence depressive episode within 1 year after treatment initiation (34.5% vs. 5.9%; OR = 8.42 [95% CI = 3.30–21.47]).

### Median time to medication noncontinuous use, mean dosage on discontinuation, and median number of clinic visits attended

The median time to noncontinuous use was 63 days. The mean dosage on discontinuation and equivalent number of DDD were listed in Table 2. The median dosage on discontinuation, as reflected by the number of DDD, was significantly higher in the SSRI group than the TCA and its related cyclic antidepressant group (1.00 vs. 0.33; *P* < 0.001), The median time to medication noncontinuous use were 46.5 and 69.5 days for TCA and its related antidepressants and SSRIs, respectively. The median number of psychiatric clinic visits attended by the continuous and noncontinuous users were 5.31 (range: 3–13) and 4.33 (range: 1–12), respectively.

**Table 2:** Mean dosage on discontinuation for different antidepressants

Class of antidepressant	Antidepressant	Mean dosage on discontinuation (mg)	Equivalent number of DDD	Range (mg)	SD
TCAs & its related AD	Amitriptyline	75.0	1.00	–	–
	Imipramine	25.0	0.25	–	–
	Mianserin	15.0	0.25	10–20	7.07
	Nortriptyline	37.5	0.50	25–50	17.68
	Prothiaden	25.0	0.17	–	–
	Trazodone	116.7	0.39	50–300	98.32
SSRIs	Citalopram	15.0	0.75	10–20	7.07
	Fluoxetine	15.6	0.78	10–40	7.51
	Paroxetine	24.2	1.21	12.5–37.5	8.17
	Sertraline	62.5	1.25	25–200	49.45
Others	Mirtazapine	25.0	0.83	15–30	7.75
	Venlafaxine	81.8	0.82	37.5–187.5	46.89

DDD, defined daily dose.

### Factors for noncontinuous use of antidepressants

#### Patient-related factors

When various patient-related factors such as age, gender, type of accommodation, drinking habit, and educational level were included for logistic regression analysis, it was found that young age, female gender, and residence in public housing estate were factors significantly associated with noncontinuous use of antidepressants (Table 3).

**Table 3:** Result of logistic regression analyses for the patient-, treatment-, and illness-related factors which were associated with noncontinuous antidepressant use

Factors	*P*-value	OR	95% CI
Patient-related factors			
Age	0.008*	0.97	0.95–0.99
Female	0.029*	2.26	1.09–4.69
Public type of accommodation	0.029*	2.10	1.08–4.10
Treatment-related factors			
Experiencing side effect within 6-month treatment	0.024*	2.27	1.12–4.63
Number of follow-ups in clinic for prescription	0.003*	0.79	0.68–0.92
Illness-related factors			
Year of diagnosis	0.034*	0.62	0.40–0.96

* *P* < 0.05.

#### Treatment-related factors

In terms of treatment-related factors, the following four parameters identified from the univariate analysis were input into the logistic regression model: (1) number of follow-ups in clinic for prescriptions, (2) number of switches, (3) newly started on TCA & its related antidepressants, and (4) experiencing side effects during 6-month treatment course per recorded in medical charts. Lower number of psychiatric clinic follow-ups attended by the patient and experiencing of side effects remained to correlate significantly with higher risks of noncontinuous use of antidepressants (Table 3). Although patients newly started on TCA & its related antidepressants seemed to reach noncontinuous use earlier than SSRI-users (median number of days to noncontinuous use: 46.5 vs. 69.5 days), the use of TCA and related antidepressant was not found to be associated with higher risk of noncontinuous use in the logistic regression model.

#### Illness-related factors

Noncontinuous use of antidepressant was more frequently observed in patients who carried a previous history of depression when compared to those newly diagnosed. Consistent results were also shown in the logistic regression model where a more recent diagnosis reduced the odds of noncontinuous antidepressant use (OR = 0.62, [95% CI: 0.40–0.96], *P* = 0.034; Table 3).

### Major reasons for noncontinuous antidepressant use

Among the 87 noncontinuous users, major reasons for noncontinuous antidepressant use identified in electronic patient records or written medical records include defaulting follow-ups (*n* = 64, 73.6%), experiencing side effects (*n* = 24, 24.6%), feeling improved in condition (*n* = 16, 18.4%), and concerns of stigma over depression (*n* = 5, 5.7%). Fourteen patients (*n* = 14, 16.1%) had self-adjusted downward the dosage of antidepressants or used the antidepressants on an as-needed basis.

## Discussion

In this study, we found that 46% of patients newly started on antidepressant treatment did not complete the treatment course of 6 months. Among the noncontinuous antidepressant users, an eightfold increase in the odds of relapse or recurrence within 1 year after treatment initiation was found. To our knowledge, this is the first study studying non-adherence and the associated risk of relapse in exclusively Asian patients who were being followed up in psychiatric setting. Most other studies conducted in Asian populations focus on the rate of non-adherence and the associated predicting factors (Yeh et al. 2008; Sawada et al. 2009; Lee et al. 2010; Shigemura et al. 2010). The only study conducted in Korea that also evaluated relapse/recurrence utilized a mixed cohort consisted of patients receiving care from primary care and psychiatric care (Kim et al. 2011). While most previous studies only evaluated prescription claims database provided by a national or insurance organization, this study evaluated both the prescription record and also the electronic and written medical records of patients to obtain a clearer picture of continuity of antidepressant use and most importantly, reported the relapse or recurrence rate in a more reliable fashion.

The rate of early noncontinuous antidepressant use within the first 30 days of treatment was 12.2% which was lower than those reported in some overseas studies. Lin et al. (1995) reported the rate of early noncontinuous antidepressant use to be 28%, whereas other studies had reported rates as high as 38.8–42.4% (Vanelli and Coca-Perraillon 2008; Sansone and Sansone 2012). The variations reported by studies may be related to the difference in definitions and measurements used for noncontinuous antidepressant use, and also the study population included. This study included only new users of antidepressants in psychiatric settings while some studies included a mixed population of primary and tertiary care (Lin et al. 1995; Claxton et al. 2000; Geddes et al. 2003; Wade et al. 2009; Kim et al. 2011; Lu and Roughhead 2012). As a few previous studies have shown, receipt of follow-up care from a psychiatrist was associated with better treatment adherence, presumably related to the better management and instruction delivered to the patient (Akincigil et al. 2007; Lu and Roughhead 2012). Our patients also did not have other significant psychiatric comorbidities mandating the use of complicated psychotropic regimen, which may have contributed to a relatively better initial adherence.

### Impact of noncontinuous antidepressant use on the relapse/recurrence depressive episode within 1-year after treatment initiation

In this study, patients who did not use antidepressants continuously for 6 months were significantly more prone to having a relapse/recurrence depressive episode within 1 year after treatment initiation. These results are fairly consistent with the results found in other studies (Melfi et al. 1998; Geddes et al. 2003; Kim et al. 2011). A study conducted in the United Kingdom enrolled patients with new episodes of depression who were not previously treated with any antidepressant during a 6-month prior period (Claxton et al. 2000). The study reported that patients who remained on antidepressants continuously for at least 120 days experienced the lowest risk of relapse or recurrence. Consistent with these findings, a systematic review of randomized clinical trials reported that continuous treatment with antidepressants reduced the odds of relapse by 69% (Geddes et al. 2003). All these results have significant implication on future long-term prognosis as earlier studies and the latest STAR*D study repeatedly demonstrated that patients who had not achieved remission at 1 year of follow up experienced more subsequent relapses (Judd et al. 1998; Pintor et al. 2004; Rush et al. 2006).

Understanding the predictors of non-adherence will allow practitioners to focus their efforts in high-risk patients and provide patients with proper advice and closer monitoring. This study had identified several patient- and illness-related factors that predict noncontinuous antidepressant use. Younger patients living in public housing estate with infrequent follow-ups were significantly associated with higher rates of noncontinuous use of antidepressants. In this study, since information regarding the patients' annual family income was not available, accommodation in public housing estate was adopted as a proxy indicator reflecting low socioeconomic status (Yu and Wong 2004). The finding of younger age and lower socioeconomic status as predictors for noncontinuous drug use echoed with a few previous studies (Hansen et al. 2004; Muzina et al. 2011; Sansone and Sansone 2012). Muzina et al. (2011) examined the adherence rates among antidepressant treated patients and found that a higher percentage of patients <65 years old were non-adherent. Similar results of poorer adherence in younger patients with bipolar disorders and schizophrenia had also been previously reported (Valenstein et al. 2006; Sajatovic et al. 2007). Higher level of overall perceived stigma at the start of treatment in younger depressive patient groups (Sirey et al. 2001a) and, as a result, refusal to accept physician's diagnosis of depression have all been postulated to be potential contributors to noncontinuous antidepressant use among younger patients (Van Voorhees et al. 2005). The problem was likely further complicated by the known Chinese culture in which misconceptions about the indication and addiction potential of antidepressants was common (Yeung et al. 2004). The documentation of 16.1% of patients attempting to use antidepressants on an as-needed basis or at self-adjusted dosages was likely an underrepresentation of the actual scenario.

Although not systematically assessed in this study, it is noteworthy that defaulting follow-ups (73.6% of noncontinuous users) and concerns of stigma over depression (*n* = 5, 5.7%) have been documented as the main reasons for noncontinuous use in our population. In fact, our findings concur with another study that suggested that Chinese Americans may feel uncomfortable taking the initiative to follow-up with their doctors even if their symptoms fail to improve or they experience significant adverse effects (Yeung et al. 2004). Meanwhile, the Surgeon General's report have identified that Asians and other minority groups tend to receive less mental health services than the Caucasians in the United States (Office of the Surgeon General (US) 2001).

The two modifiable risk factors to noncontinuous antidepressant use we identified were the number of follow-ups in clinic and side effects experienced by the patient. This finding was in line with a prospective randomized controlled trial conducted by Katon et al. (1995). Katon et al. (1995) reported that patients receiving increased intensity and frequency of visits over the first 4–6 weeks of treatment (visits 1 and 3 with a primary care physician, and visits 2 and 4 with a psychiatrist) had significantly greater drug adherence for 90 days or more (75.5% vs. 50.0%). A similar association of number of follow-ups in clinic for prescriptions with antidepressant continuation was found in another study, which showed that more than three follow-up visits for depression was associated with less discontinuing use of antidepressants (OR: 0.40, 95% CI: 0.19–0.82; Bull et al. 2002). Therefore, increasing frequency of the follow-ups with focus to manage expectation on side effects and de-stigmatization over depression should be explored as a way to improve the noncontinuous use. These highlighted the importance of the need for systematic psychoeducation on the depressive illness and reinforcement of patients' drug adherence as suggested in another local study conducted in patients with mood disorders (Lee et al. 1992).

### Limitations

While this study evaluated both the prescription record and also the electronic and written medical records of patients, limitations related to retrospective data retrieval still apply when interpreting our findings. The use of retrospective data retrieved from the prescription database and medical records may have underestimated the rate of noncontinuous use. As the data relied on patient reporting and prescription filling, it does not reflect the actual drug use of the patients. The sample size was relatively modest compared to previous studies using only claims database as source of data. However, the difference in relapse rate between the continuous and noncontinuous users was highly significant. Therefore, it is unlikely that our modest sample size critically affected our results and conclusions, although it is possible that not all relevant predictors for noncontinuous use were identified. Meanwhile, the exclusion of comorbid diagnosis or a past history of suicide may have potentially excluded a group of most severe depressive patients. Some studies have previously suggested an impact of concurrent use of benzodiazepine on the continuity of antidepressants, but this is beyond the scope of this study (Morgan et al. 2011)^.^ Finally, the unavailability of standardized, quantitative measurement for defining disease severity, relapses or remission was also one of the limitations in this study.

Noncontinuous antidepressant use is an important predictor of relapse and recurrence with significant implication for long-term prognosis. The results found in this Chinese population highlighted the high early recurrence rate. Collaborative multidisciplinary approach utilizing various health care professionals to provide systematic psychoeducation on depressive illness and drug aspects should be explored.
